# Temperature effect on the nucleation of graphene on Cu (111)†

**DOI:** 10.1039/c8ra05478a

**Published:** 2018-08-03

**Authors:** Behnaz Rahmani Didar, Homa Khosravian, Perla B. Balbuena

**Affiliations:** Artie McFerrin Department of Chemical Engineering, Texas A&M University College Station Texas 77843 USA hkhosravian@tamu.edu

## Abstract

Repeated thermal cycling by using an organic precursor is shown to be a successful technique for growing graphene on metal substrates. Having control on this process is of vital importance in producing large areas of high quality graphene with well-ordered surface characteristics, which leads us to investigate the effect of temperature on the microscopic mechanisms behind this process. Apart from being an important factor in the dissociation of the organic precursor and promoting the reactions taking place on the surface of the catalyst, temperature also plays a major role in the structure of the catalyst surface. First, we used eight thermal cycles to successfully grow graphene on the surface of Cu (111). Then, we employed *Ab Initio* Molecular Dynamics (AIMD) simulations to study graphene island alignment evolution at two temperatures. The results shed light on our experimental observations and those reported in the literature and point to the effectiveness of controlled thermal cycling in producing high quality graphene sheets on transition metal catalyst surfaces.

## Abstract 2

Repeated thermal cycling by using an organic precursor is shown to be a successful technique for growing graphene on metal substrates.

## Graphical abstract

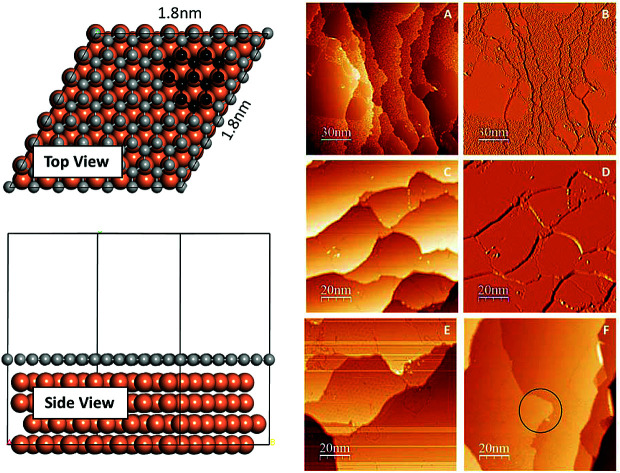

## Introduction

Graphene, a single atomic layer of graphite and the building block of carbon nanotubes, has been proven to possess many unusual properties such as extraordinary chemical and optical characteristics, tensile strength and thermal and electrical conductivity that find applications in many areas such as ultrathin membranes, plasmonics, high-end composite materials and electronics.^1–7^ Since its discovery, graphene has been synthesized through several methods including graphene oxide reduction (chemical synthesis method), mechanical exfoliation from graphite and hydrocarbon dissociation on transition metal substrates (chemical vapor deposition, CVD). Of these methods, mechanical exfoliation, although capable of producing high quality graphene on small scales, is the least scalable and appropriate method for electronics applications and mass production where larger areas of graphene are required, whereas the CVD method, typically catalytic, has emerged as the most powerful and dominating method in the industry.^8,9^

Ni, Ir and Cu are three of the most common transition metals that are used as catalyst substrate for graphene growth.^3^ Growth mechanism on these substrates are shown to be highly dependent on temperature.^10^ Among these substrates, copper has become one of the most widely used catalysts for the synthesis of graphene using the CVD method. Large high-quality graphene films were grown on copper, which exhibit transferability and unique plasmonic characteristics.^2,11,12^ Low carbon solubility^13,14^ and weak Cu–C interactions^15,16^ along with low surface diffusion energy barrier of C atoms on Cu^17^ all contribute to surface-driven mechanisms present in the nucleation and growth of graphene domains.^3,18–20^ These factors may also explain why Cu produces predominantly single layer graphene.^3,21^ Low surface diffusion energy barrier contributes to highly mobilized carbon atoms that can travel freely on the Cu surface and attach to existing graphene islands. Note that surface Cu atoms have high mobility at high temperatures which can facilitate the mobility of C atoms and graphene islands at such temperatures and lead to defect healing.^20,21^ Therefore, surface morphology of Cu substrate at elevated growth temperatures is an important issue.

Reported research aimed at improving the quality of graphene grown over Cu surface were focused on several aspects of this process. As an example, Wassei *et al.* investigated how the type of organic precursor may have effect on graphene growth and dictate its structure.^22^ Ethylene, acetylene and methane are organic precursors that are widely used as the source gas of which methane is known to require higher temperatures to decompose on the Cu surface. Ethylene^23^ and acetylene,^24^ on the other hand, has shown to produce continuous graphene coverage at considerably lower temperatures. In other studies, environmental conditions such as temperature, pressure and hydrogen that may have effect on graphene growth were investigated.^10,25,26^ The copper catalyst surface has also been subject to many studies as the surface plays a major role in the epitaxial graphene growth and achieving a perfectly clean surface prior to growth is highly desirable.^27–30^ Yu *et al.* reported their simple approach of preparing the copper surface by growing it on single-crystal sapphire. The resulting copper layer is reported to be extremely flat, chemically clean and can be easily peeled off to use for graphene growth. The graphene grown on such a copper surface exhibited very large domain sizes.^31^

High resolution electron microscopy provides valuable insights into defects and domain (grain) boundaries in graphene produced from the CVD method. These domain boundaries have been subject to intense studies and may hold the key to understanding the growth mechanism. Two main mechanisms have been proposed for the growth of graphene on transition metal surfaces: segregation growth (Ni, Ru, Ir), and surface growth (Cu).^3,18^ In the surface-driven mechanism of growth on copper, it is shown that graphene islands grow when temperature is high enough to facilitate the diffusion of islands. At such temperatures some islands may ‘stitch’ together to form larger graphene domains.^32–36^ Consequently, the overarching hypothesis is that defects observed in the graphene overlayer arise from the misalignment of graphene islands constituting a domain; various islands nucleate and grow with different rotational alignments across the catalytic substrate. Several studies have been aimed at investigating what dictates these island orientations. Studies in this area have concluded that domain shapes and sizes themselves are controlled by growth conditions such as temperature.^37,38^ Upon closer examination, two possible routes have been identified that would lead to the transition of islands to domains; either the islands migrate and coalesce into larger islands, or islands progressively grow in size and form domains through addition of C atoms.^35,39,40^ In the former, with the foregoing discussion, temperature would have great influence on rotation and migration of islands and domain growth, as well as on nucleation density, as evidenced earlier.^38,41^ While in the latter case, surface diffusion of C adatoms would be most likely to have a greater impact on growth of domains. Thus, the identification of the prevailing case will help in optimizing growth parameters. Although this may be a challenge since many factors are involved in the competition including carbon source gas decomposition rate, nucleation rate, surface diffusion and growth rate from carbon adatom addition.

In this paper, our particular interest is to understand the role of nucleation and growth parameters, such as temperature, on graphene domain orientation. In this regard, the catalyst surface may have a more active role than thought in the determination of domain boundaries. In the present work, using experimental and *ab initio* methods, we investigate how temperature and time cycles (annealing and source gas exposure cycles) may affect the alignment between the Cu lattice and a pre-existing graphene fragment. Our focus is to study the interplay of the Cu substrate and the graphene overlayer in conjunction with temperature at early stages of growth.

## Experimental details

All the experiments were carried out in an ultrahigh vacuum system (base pressure approximately 1 × 10^−10^ mbar) equipped with a variable temperature scanning tunneling microscope (VT-STM, Omicron NanoTechnology GmbH, Taunusstein, Germany) chamber, and a separate preparation chamber, which includes an ion sputter gun for sample cleaning, a LEED (Low Energy Electron Diffraction) system, and a directional gas doser. The Cu (111) single crystal (Marketech International, Port Townsend, WA) was polished on one side and mounted on a standard tantalum sample plate from Omicron with an 8 mm diameter hole on its rear end to facilitate electron bombardment heating. Cu (111) was cleaned by 8 cycles of sputtering (4 × 10^−6^ mbar Ar, 1.0 keV, 1 μA, 20 minutes) and annealing (1000 °C, 10 minutes, *in vacuo*). Temperature above 600 °C (873 K) was monitored by a pyrometer (model OS3708; Omega Engineering Inc., Stamford, CT). Successful cleaning of the sample was confirmed by STM and LEED. Large continuous graphene islands were produced on Cu (111) surface through exposing the clean Cu (111) substrate to 1 × 10^−5^ mbar C_2_H_4_ at room temperature for 5 minutes, with subsequent annealing at 1000 °C (1273 K) for 5 minutes. This cycle was repeated 8 times.^64^ Successful graphene preparation was confirmed by scanning tunneling microscopy (STM). All STM images were collected at room temperature by using electrochemically etched W homemade tips with a sample bias of 0.7 V and a constant tunneling current of 0.4 nA. All the post annealing images were generally acquired 2 hours after the experiment to ensure that the sample had returned to room temperature. To ensure that the images shown here are consistent across the crystal face, for any given surface condition, images were collected at multiple locations (usually 4 or 5) on the surface. All the STM data were processed using WSxM 5.0 software.^42^

## Computational details

**Fig. 1:**
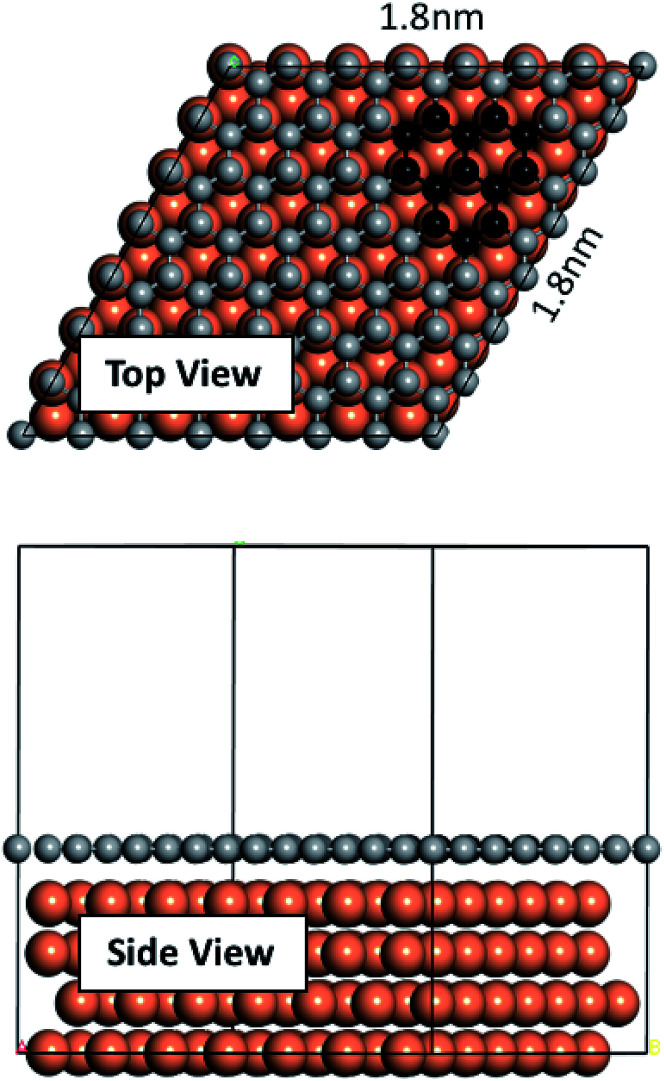
Cu slab used in this work and the single graphene overlayer from which the fragments were extracted.

We performed our density functional theory (DFT) calculations using the Vienna Ab initio Simulation Package^43–45^ with Perdew–Burke–Ernzerhof (PBE) exchange-correlation functional and in the generalized gradient approximation (GGA) formulation.^46^ We treated the electron-ion core interactions using the projector-augmented wave (PAW) pseudopotentials^45,47^ available in the VASP database. We constructed a four atomic layer thick *p* (7 × 7) Cu slab in Materials Studio^48^ by cleaving along the (111) plane. A vacuum of 20 Å thickness was applied on the slab along the *z* direction to prevent interactions of the slab with its upper/lower periodic images. The bottom layer was kept fixed and the remaining three layers were allowed to relax. Conjugate gradient algorithm with a Gaussian smearing width of 0.05 eV was used for all relaxations. Convergence tests were carried out, and a 1 × 1 × 1 *k*-point mesh with a plane wave energy cutoff of 400 eV was selected for the sampling of the Brillouin zone. The graphene fragments used in this study consist of three neighboring hexagonal rings. Graphene fragments were initially taken from Materials Studio structures database and then relaxed. Relaxed structures were then used on the Cu surface. Fig. 1 depicts the constructed Cu slab and a single graphene overlayer of which the graphene fragments were extracted. *Ab initio* molecular dynamics (AIMD) were performed using VASP with the foregoing computational details. AIMD simulations were carried out in the NVT ensemble with a 1 fs time step and at two temperatures of 900 K and 1200 K.

## Results and discussion

### Experimental observations

**Fig. 2:**
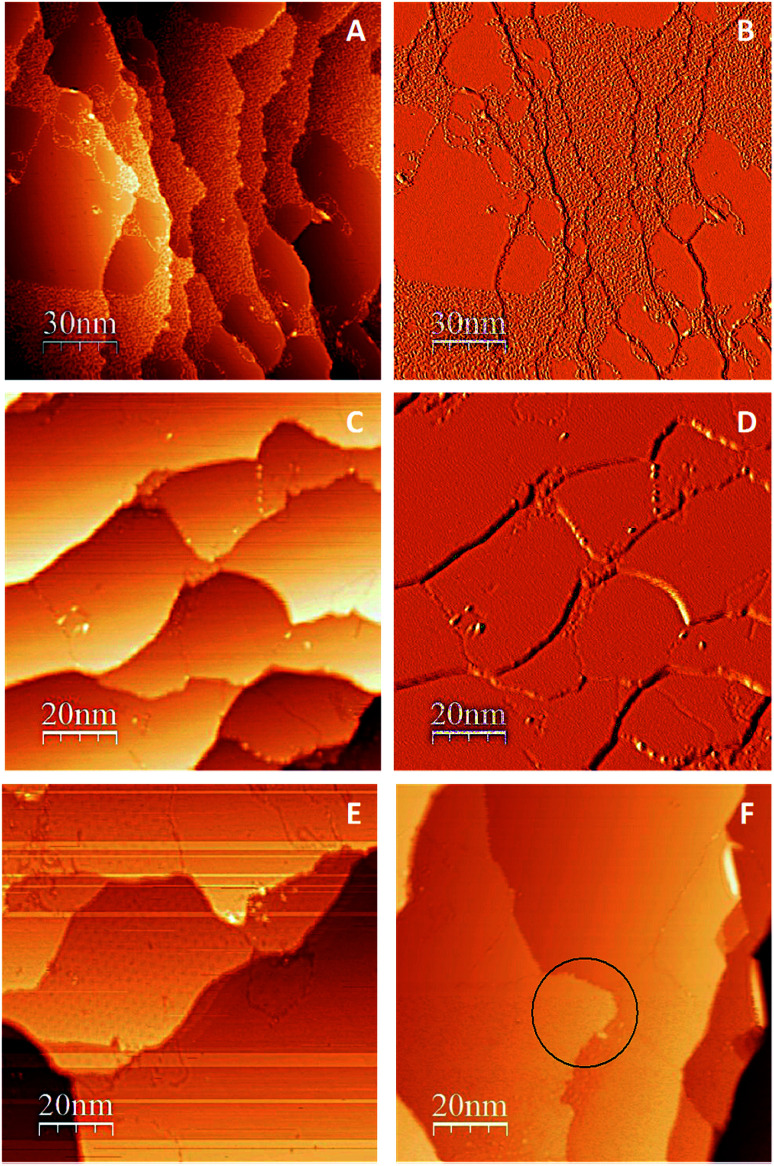
Topographical and differential conductance (d*I*/d*V*) STM images of graphene growth on Cu (111). (A) Graphene islands and C atoms are both observed in one single image before final controlled annealing. (B) Differential conductance (d*I*/d*V*) image of the STM image shown in A. Graphene islands look smoother and darker in differential conductance images. (C) Complete graphene coverage over Cu (111) after final annealing. The domain boundaries are visible in this STM image. (D) Differential conductance (d*I*/d*V*) image of STM image shown in C. The entire image has the same color, which indicates either all the terraces are Cu (111) or graphene/Cu (111). Other parts will support that the latter is true. (E) Moiré pattern was observed on some terraces. (F) Graphene films grown through the controlled thermal cycling method terminated at Cu (111) step edges but changed their shape (the area inside the black circle shows such a shape change).

One promising approach to prepare large graphene sheets with low defects on metal substrates is by thermal dissociation of an organic precursor on the metal surface.^49–54^ Gao *et al.* showed that epitaxial growth of graphene on Cu (111) by thermal decomposition of ethylene (C_2_H_4_) at 1000 °C results in formation of single-layer graphene platelets and sheets.^33^ Similarly, to produce large continuous graphene islands on Cu (111) surface, we undertook a controlled thermal cycling approach using the organic precursor, (C_2_H_4_). Successful preparation of large continuous graphene film over Cu (111) surface was confirmed by STM as shown in Fig. 2. Since different moiré patterns could be observed under specific tunneling conditions, it was difficult to capture all moiré patterns in one single image. Thus, more than one method was needed to interpret the STM data and to conclude whether we had a full coverage graphene/Cu (111). Fig. 2A (topographical image), and Fig. 2B (differential conductance (d*I*/d*V*) image) were captured after the 7^th^ cycle of thermal annealing. As shown in these two images, graphene islands and C atoms are both observed in one single image before final controlled annealing. Note that graphene islands look smoother and darker in differential conductance images. However, as shown in Fig. 2C (topographical image) and Fig. 2D (differential conductance (d*I*/d*V*) image), after final cycle of thermal dissociation of C_2_H_4_ (8^th^ cycle), complete graphene coverage over Cu (111) was observed. The domain boundaries are visible in these STM images. Note that the entire differential conductance image has a uniform color, which indicates that either all the terraces are Cu (111) or graphene/Cu (111) and Fig. 2E and F support that the latter is true. Moiré pattern was observed on some terraces of Fig. 2E and F shows the shape changes of Cu (111) terrace, which is a result of successful preparation of graphene.^49^ As shown in Fig. 2E and F, graphene films grown through this method terminated at Cu (111) step edges but changed their shape (the area inside the black circle in Fig. 2F shows such a shape change).

### Adsorption of C_6_ rings and graphene islands on Cu (111)

Next we focused on a first-principles analysis of the graphene growth mechanisms. The adsorption of C_6_ rings and graphene islands on different adsorption sites has been studied elsewhere using DFT.^4,55^ Compared to these earlier studies, the cell used in this work is larger to accommodate end effects, and a GGA functional used (PBE) which is more accurate than the local density approximation (LDA). Using DFT optimizations, we allowed a C_6_ ring to relax onto the Cu (111) surface. We tested three main possibilities; (a) the ring surrounds a top Cu atom and the C atoms of the ring occupy fcc and hcp hollow sites (fcc–hcp), (b) the ring surrounds an hcp site and the C atoms occupy fcc and top sites (fcc-top), and (c) the ring surrounds an fcc site and the C atoms occupy hcp and top sites (hcp-top), alternately. Results, illustrated in Fig. S1 of ESI,† showed that the fcc–hcp is the most thermodynamically favorable site. This arrangement also allows for perfect matching of the Cu (111) lattice and the honeycomb structure of the hexagonal ring, whereas other arrangements cause slight stress in the hexagonal ring. Also inferred from the figure is that the adsorption energy differences between the possible adsorption sites are less than 0.05 eV per C atom. Furthermore, Fig. S2 and S3† illustrate the energy barriers obtained from NEB calculations for the diffusion of the fcc–hcp arrangement to the fcc-top and hcp-top. These barriers are approximately 0.45 eV which is quite low. Therefore we can conclude that a single hexagonal ring, once formed, can travel on the Cu surface at a low energy cost. The perfect match between the ring and Cu lattice is only limited to a small number of rings. With an increase in the number of rings, the mismatch between the lattices of graphene and Cu will eventually lead to moiré patterns. This is highlighted in Fig. S4 of ESI,† and revealed by the experimental characterization shown in Fig. 2E.

In addition, we also allowed a pre-relaxed graphene fragment, composed of ^13^C atoms constituting 3 rings, to relax on the Cu surface. We studied 7 different orientations of the fragment with respect to the Cu lattice. These orientations were from 0° to 60° with 10° increments. Results are illustrated in Fig. S5 and S6 of ESI.† The energy required for the adsorption of various rotations is slightly greater than a single ring. Nevertheless, similar to a single hexagonal ring, a strong preference towards a certain alignment of a graphene fragment with Cu is not apparent, although the 40° is slightly less favorable than others. The most energetically favorable graphene orientation on Ir (111), Au (111) and Cu (111) has been identified and reported elsewhere.^38,56,57^ Here, our focus was to determine the most favorable orientations of a graphene fragment rather than a graphene sheet on the Cu surface. This represents the initial nucleation stages observed experimentally on the Cu (111) terraces as discussed in relation to Fig. 2.

### Effect of temperature on the rotation and orientation of an isolated fragment

**Fig. 3:**
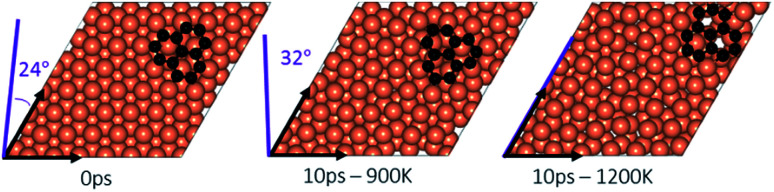
An isolated graphene fragment adsorbed on the Cu (111) surface before AIMD (0 ps) and after 10 ps at temperatures of 900 K and 1200 K. The initial orientation (24°) of the fragment with respect to Cu lattice is shown in purple at 0 ps. The final orientations are 32° for 900 K and zero for 1200 K.

**Fig. 4:**
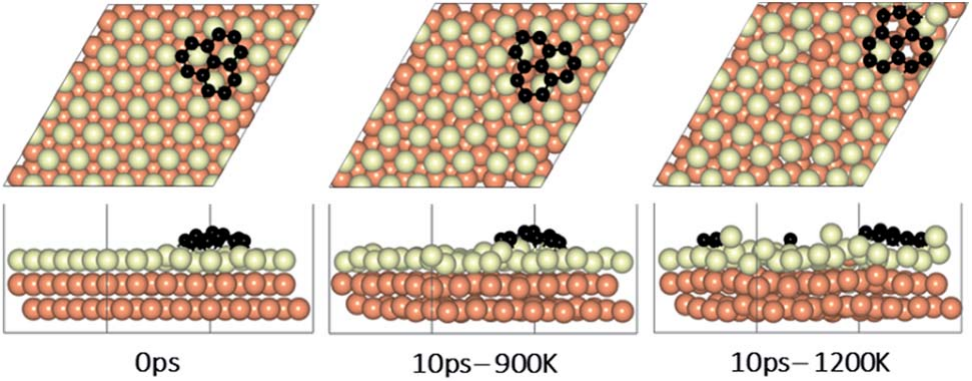
Surface reconstruction observations of the Cu slab in the presence of a graphene fragment taken from 10 ps of AIMD simulations performed at temperatures of 900 K and 1200 K. Light green atoms depict surface Cu atoms.

In the experimental part of this work, the technique of low and high temperature cycles was applied to grow the graphene sheet. Reportedly, graphene coverage can be controlled by the number of thermal cycles. Here we emulated several aspects of this process by using AIMD simulations to understand the effect of these cycles on the stability of the graphene fragments with respect to the underlying substrate. First, we studied the motion and rotation of an isolated fragment at two temperatures of 900 K and 1200 K. The selection of temperatures was largely dictated by other experimental studies^33^ as well as our own. Prior to running AIMD simulations, the fragment was relaxed onto the Cu surface with an initial misalignment of 24° with respect to the Cu (111) lattice as shown in Fig. 3. The relaxed system was then allowed to evolve for 10 ps while rotation and alignment of fragment was checked every 1 ps. The evolution is illustrated in more detail in Fig. S7 and S8 of ESI.† Clearly, the fragment does not remain stationary but is rather in coordinated motion with the underlying surface Cu atoms as they reorder and reconstruct the surface (shown in Fig. 4, S13 and S14†). We observed the fragment to rotate in both clockwise and anti-clockwise directions. At the higher temperature of 1200 K and within 10 ps, the fragment had rotated to the extent of completely aligning with the Cu (111) lattice. At the lower temperature of 900 K, the same relaxed fragment was less mobile and was not able to correct the initial 24° misalignment with the Cu lattice. What is apparent from the images is that the surface Cu atoms are more disordered at the higher 1200 K temperature. The ability of the graphene fragment to correct the misorientation at the higher temperature of 1200 K may therefore be linked to the elevated mobility of surface Cu atoms at higher temperatures.

### Effect of temperature on the rotations and alignments of a pair of fragments

**Fig. 5:**
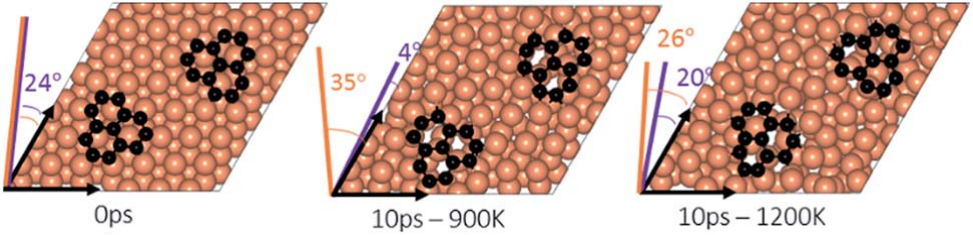
A pair of graphene fragments adsorbed on the Cu (111) surface (distance between centers of mass = 1 nm) before AIMD (0 ps) and after 10 ps at temperatures of 900 K and 1200 K. The initial orientations (24°) of both of the fragments with respect to Cu lattice is shown in purple at 0 ps. The final orientations at 900 K are shown as 35° for the left fragment (in orange) and 4° for the right (in purple). The final orientations at 1200 K are 26° for the left fragment (in orange) and 20° for the right (in purple).

**Fig. 6:**
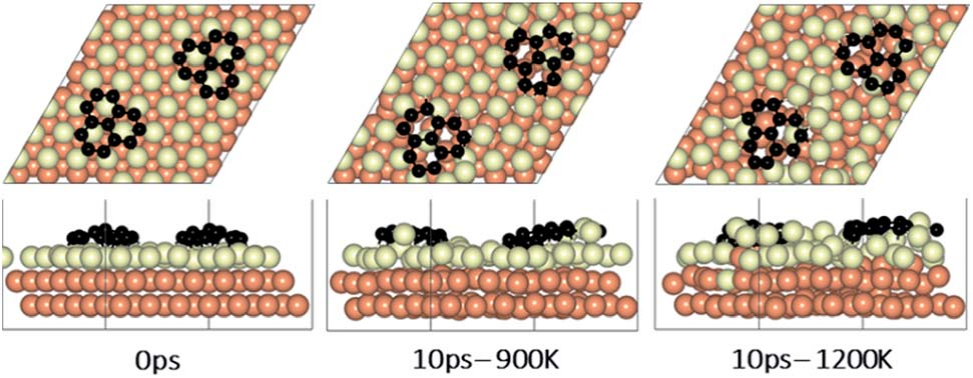
Surface reconstruction observations of the Cu slab in the presence of a pair of graphene fragments taken from 10 ps of AIMD simulations performed at temperatures of 900 K and 1200 K. Light green atoms depict surface Cu atoms.

To understand the effect that neighboring graphene fragments may have on a single fragment, we added a second fragment to the vicinity of the isolated fragment of the previous section. We note that the orientation of the second fragment with the first fragment was not set arbitrarily. We tried four different orientations of 0°, 30°, 60° and 90° between the two fragments. The most energetically favorable was the 60° orientation. This structure resulted in 24° misalignment of both fragments with the Cu (111) lattice and we started the AIMD simulations with this initial structure. Fig. 5 shows results of AIMD simulations. Detailed evolution of fragments rotations and alignments can be found in Fig. S9 and S10 of ESI.† As in the isolated fragment, we observed rotations of fragments in both directions. Rotations of the two fragments were also observed to oppose each other. At the higher temperature of 1200 K and at the end of 10 ps, we observed that neither of the fragments were able to align with the Cu lattice. The disordering of the surface Cu atoms, shown in Fig. 6, was also quite significant. At the lower temperature of 900 K, the misalignments with Cu lattice were less and one of the fragments succeeded in gaining close alignment with the Cu lattice. As evident from the figures, surface reconstruction of Cu atoms is low at this temperature. Therefore, while the higher 1200 K temperature was effective in mobilizing surface Cu atoms and aligning the isolated fragment with the Cu lattice, in the presence of a second fragment, this surface disordering is even more intense and may perhaps hinder the correction of the misalignment. Therefore, we find that once small graphene fragments are formed, the more moderate temperature of 900 K, may be more effective in achieving alignment with the Cu lattice. More details of surface reconstructions can be found in Fig. S15 and S16 of ESI.†

### Effect of fragments proximity on the rotation and alignments of fragments

**Fig. 7:**
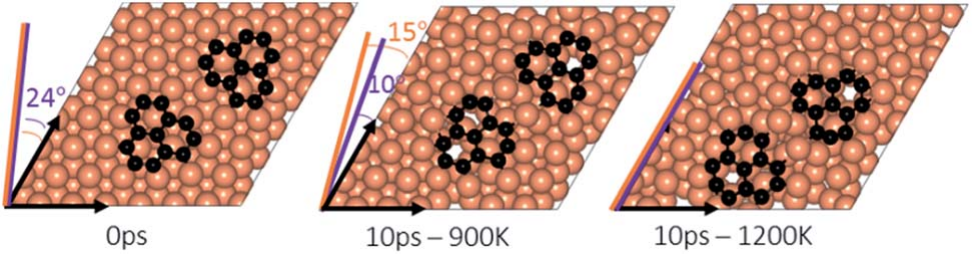
A pair of graphene fragments adsorbed on the Cu (111) surface at close vicinity to each other (distance between centers of mass = 0.8 nm) and after 10 ps at temperatures of 900 K and 1200 K. The initial orientations (24°) of both of the fragment with respect to Cu lattice is shown in purple at 0 ps. The final orientations at 900 K are 15° for the left fragment (in orange) and 10° for the right (in purple). The final orientations of both fragments at 1200 K are zero.

**Fig. 8:**
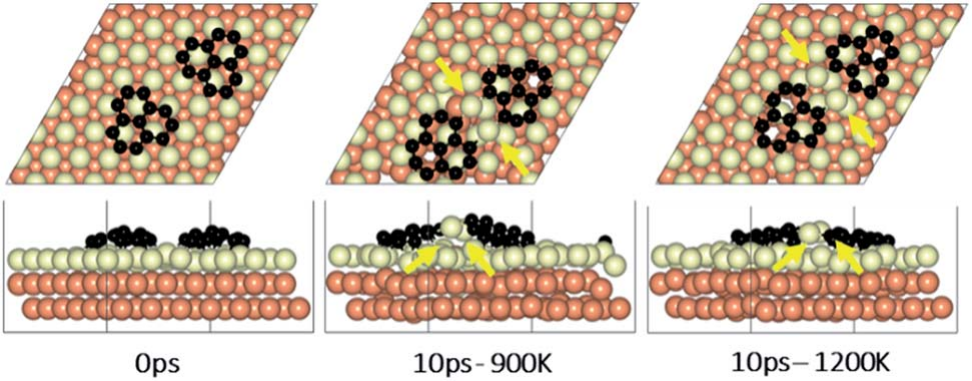
Surface reconstruction observations of the Cu slab in the presence of a pair of graphene fragments at close vicinity taken from 10 ps of AIMD simulations performed at temperatures of 900 K and 1200 K. Light green atoms depict surface Cu atoms.

To study the effect of proximity on additional graphene islands in the vicinity of the first one, we placed the second graphene fragment of the previous section, closer (by 0.2 nm) to the initial fragment and relaxed the structure before running AIMD simulations. Results are shown in Fig. 7 and with more details in Fig. S11 and S12 of ESI.† As such, at 1200 K, the pair fragments were able to align with the Cu lattice. In the majority of times, the two fragments rotated in the same direction. At 900 K, however, the misalignment decreased but persisted throughout the 10 ps of simulation time. The Cu surface reconstruction (shown in Fig. 8, S17 and S18†) was also considerably lower than when fragments were farther away from each other. The greater degree of disorder seen in the case of fragments farther apart from each other is in fact the surface Cu atoms being raised with unsuccessful attempts to create bridges between the two fragments. Note that this is a periodic cell. Considering neighboring images to the depicted two fragments, the raising of surface Cu atoms is occurring in all directions. However, the fragments are not as close for bridging to occur. When the fragments are in close proximity, the bridging is achieved on the side that fragments are closer to each other, while the remaining area in the cell is not close enough to any fragment (even in periodic images) for surface Cu atom to rise.

The bridging-metal structure previously reported for Cu,^58–60^ Fe^61^ and Ni^61,62^ surface atoms in graphene growth was clearly observed in this part of the work. Both fragments were able to bond to two surface Cu atoms situated between the two fragments and by doing so, raise those atoms from the surface (seen in Fig. 8). Inevitably, the two metal atoms bridging between two graphene fragments, lead to the equaling of fragments orientations with respect to each other and unifying their rotations and movements on the Cu surface. In comparison with the case of two fragments farther away from each other, graphene fragments located in close proximity of each other, *i.e.* high density of graphene fragments on the Cu surface, seems to accentuate the ability of higher temperatures to correct graphene/Cu misalignment. Once greater number of graphene fragments/islands are formed at lower temperatures, increasing the temperature, may help coalesce the fragments into a larger fragment that is well-aligned with respect to the Cu lattice. We suspect that at higher graphene island densities, these bridging metal atoms will eventually be suppressed down to the rest of the metal catalyst surface as graphene islands are brought into contact with each other. This hypothesis can be observed in the STM images (Fig. 2). The density of independent graphene fragments is large enough just before the final annealing after which a complete graphene layer is formed. The graphene layer does not show major moiré patterns and holds a certain alignment with the Cu substrate. The moiré patterns observed are especially located on some terraces. This observation was also reported elsewhere.^33,63^

## Conclusions

In this work, we studied the effect of temperature and thermal cycles on the alignment of graphene islands in the early stages of epitaxial growth of graphene on Cu(111) surface when only small graphene islands have been formed. Hereafter, the islands may align with respect to one another and form a uniform graphene sheet or each may grow about their own specific alignment with the Cu lattice and produce various domains. We closely examined the effect that temperature may have in choosing either of these paths. We found that higher temperatures generally lead to the alignment of an island on the Cu surface. An isolated graphene pallet at high temperatures (above 900 K) is able to correct an initial misalignment and align itself perfectly with the Cu surface. This is attributed to the higher mobility of the Cu catalyst surface layer which allows graphene to rotate freely. As growth proceeds at lower temperatures and the number of graphene fragments increases, metal-bridging of surface Cu atoms which was observed unifies graphene islands. Therefore, increased growth rate at lower temperature combined with increased mobility at higher temperatures lead to production of large high quality graphene films with the perfect alignment of graphene islands with Cu lattice.

## Conflicts of interest

There are no conflicts to declare.

## Supplementary Material

RA-008-C8RA05478A-s001
