# Supplementary material for: Temperature effect on the nucleation of graphene on Cu (111)
Source: RSC Adv. 2018 Aug 3;8(49):27825–31. doi: 10.1039/c8ra05478a (PMC9083936; doi:10.1039/c8ra05478a)
Supplement: RA-008-C8RA05478A-s001 [file RA-008-C8RA05478A-s001.pdf]

Electronic Supplementary Material (ESI) for RSC Advances.
This journal is © The Royal Society of Chemistry 2018

Supporting Information

Department of Chemical Engineering, Texas A&M University, College Station, Texas 77843

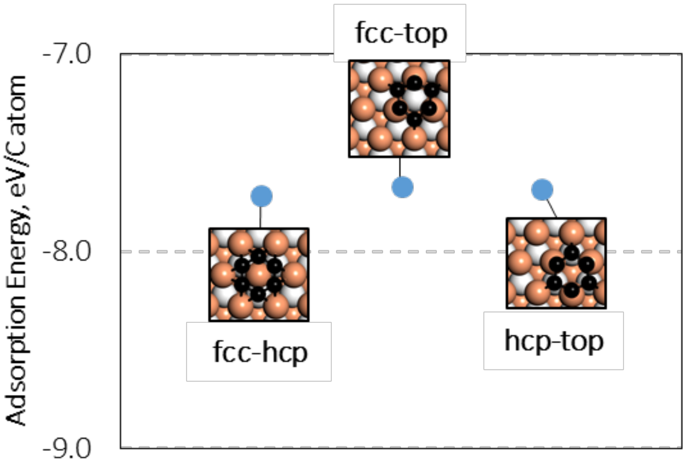

Figure S1. Adsorption energies and structures of three possible adsorption sites for a C6 ring on the Cu (111) surface. Top and second layer Cu atoms are colored in orange and white, respectively. Differences between adsorption energies are within 0.05eV/C from one another. The adsorption energies are calculated by:

$$E_{ads} = \frac{E_{sys} - E_{Cu\,Slab} - n_C E_C}{n_C(= 6)}$$

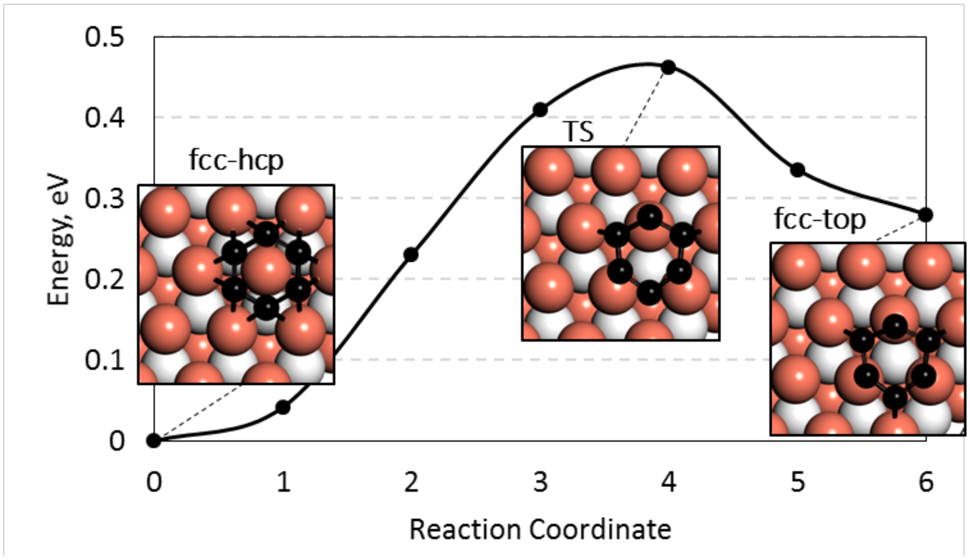

Figure S2. Diffusion of a C6 ring on the Cu (111) surface from fcc-hcp site to fcc-top site obtained from NEB DFT calculations. Initial (fcc-hcp), TS (transition state) and final (fcc-top) images are also depicted. Top and second layer Cu atoms are colored in orange and white, respectively. Energy barrier for forward and reverse reactions are 0.46 eV and 0.18 eV, respectively.

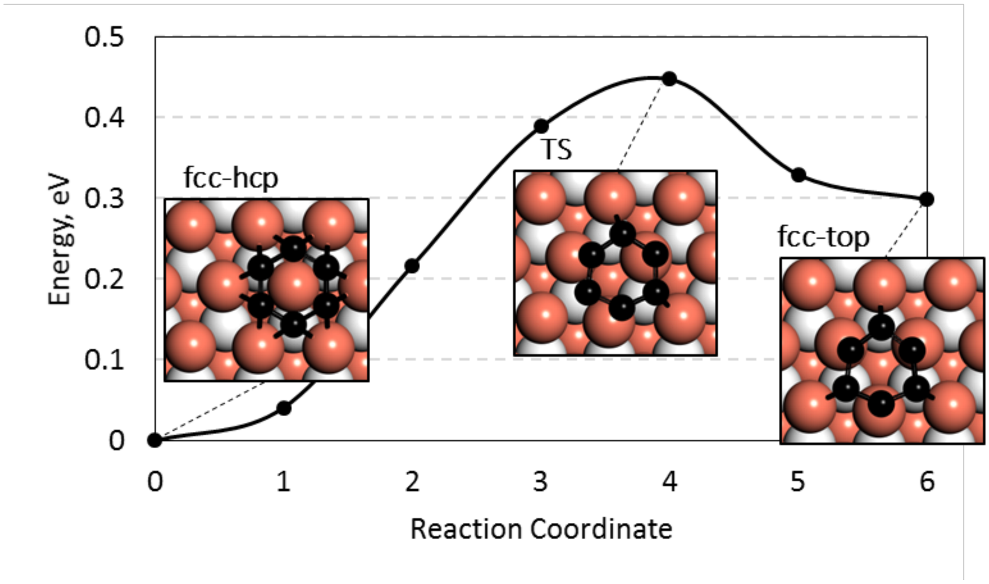

Figure S3. Diffusion of a C6 ring on the Cu (111) surface from fcc-hcp site to hcp-top site obtained from NEB DFT calculations. Initial (fcc-hcp), TS (transition state) and final (hcp-top) images are also depicted. Top and second layer Cu atoms are colored in orange and white, respectively. Energy barrier for forward and reverse reactions are 0.45 eV and 0.15 eV, respectively.

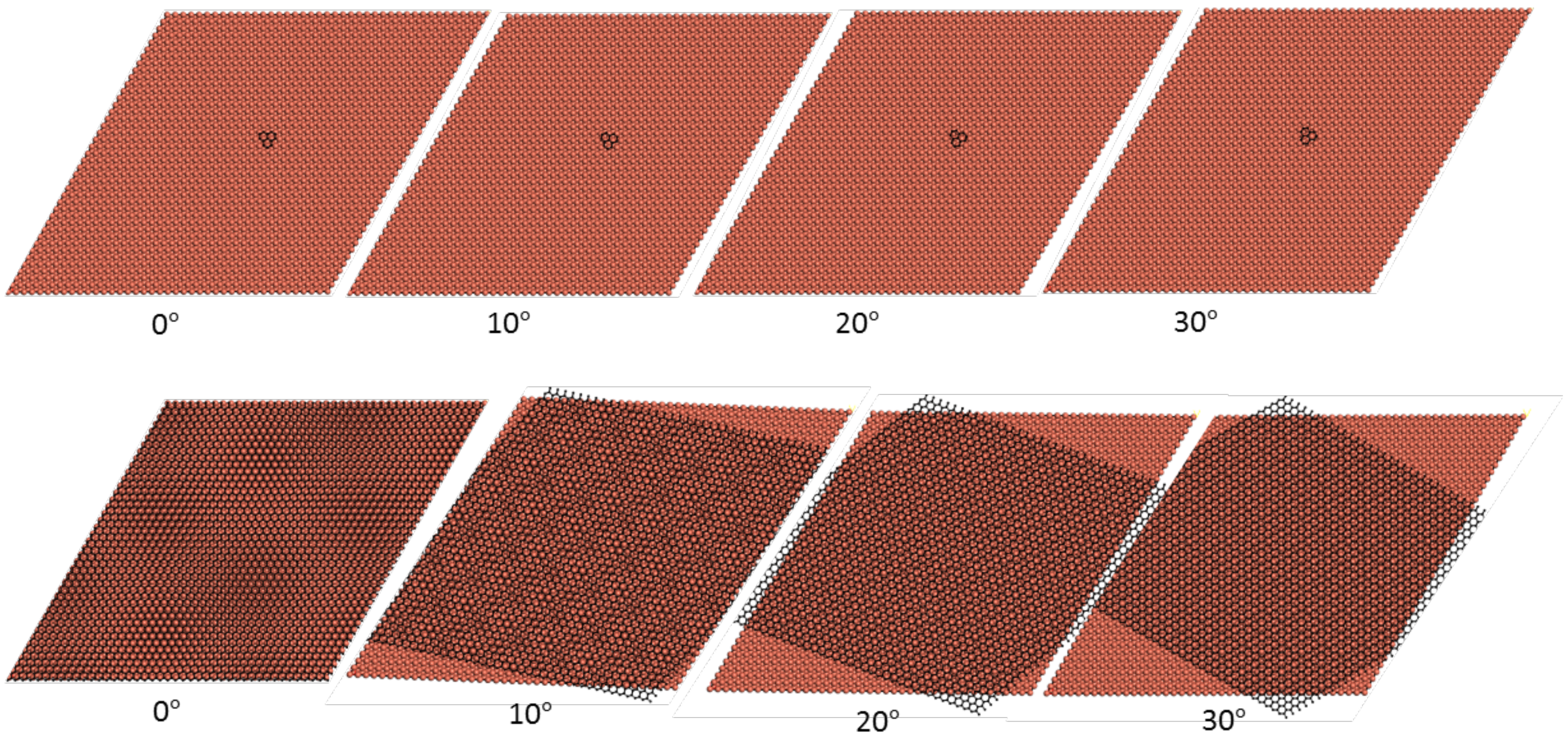

Figure S4. 4 different orientations (0°, 10°, 20° and 30°) of Top) a graphene island consisting of 3 hexagonal rings and bottom) a graphene sheet, with respect to the underlying Cu (111) lattice. Unlike the top images, Moiré patterns are clearly visible in the bottom images.

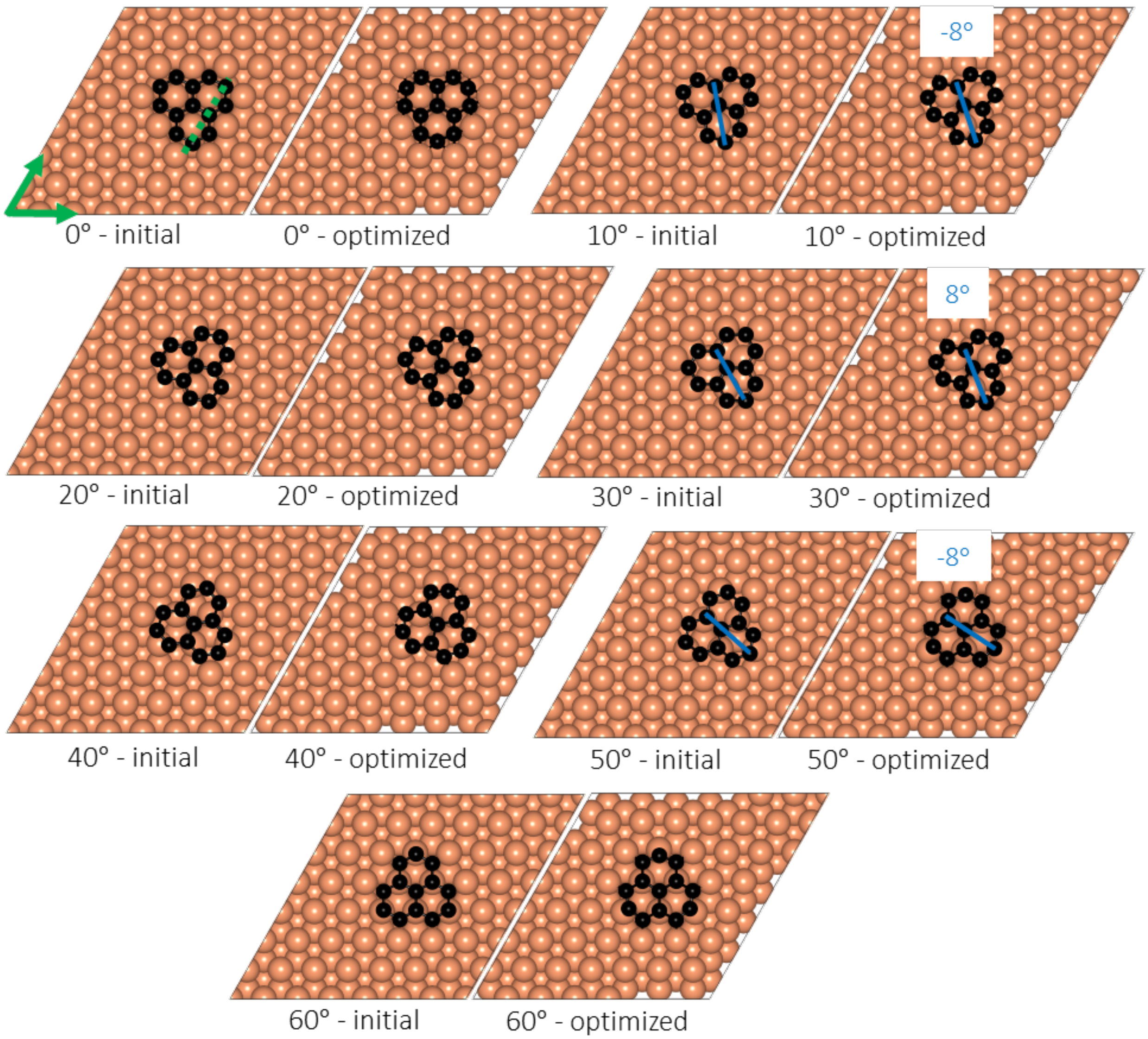

Figure S5. Rotation of a graphene fragment on the Cu (111) surface upon geometry optimization of initial alignments of 0° to 60° with the Cu lattice. Negative values correspond to anti-clockwise rotation.

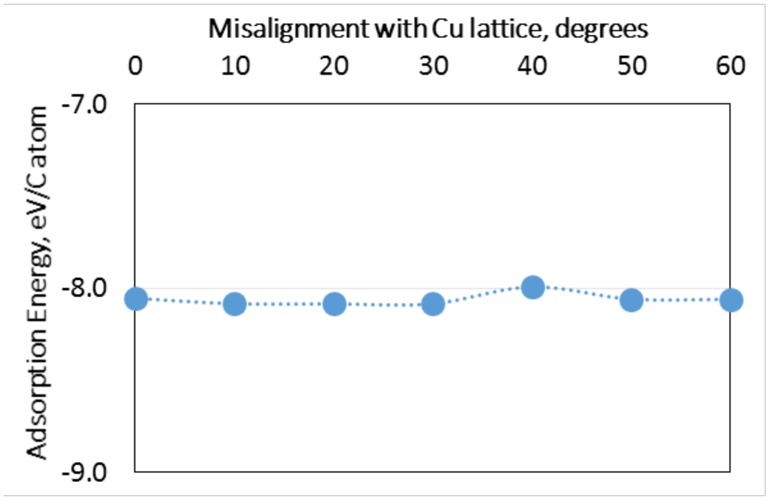

Figure S6. Adsorption energies per C atom referring to the graphene fragment on the Cu (111) with initial alignments of 0° to 60° with the Cu lattice. Differences in adsorption energies are within 0.1eV/C from one another. The adsorption energies are calculated by:

$$E_{ads} = \frac{E_{sys} - E_{Cu\,Slab} - n_C E_C}{n_C(= 13)}$$

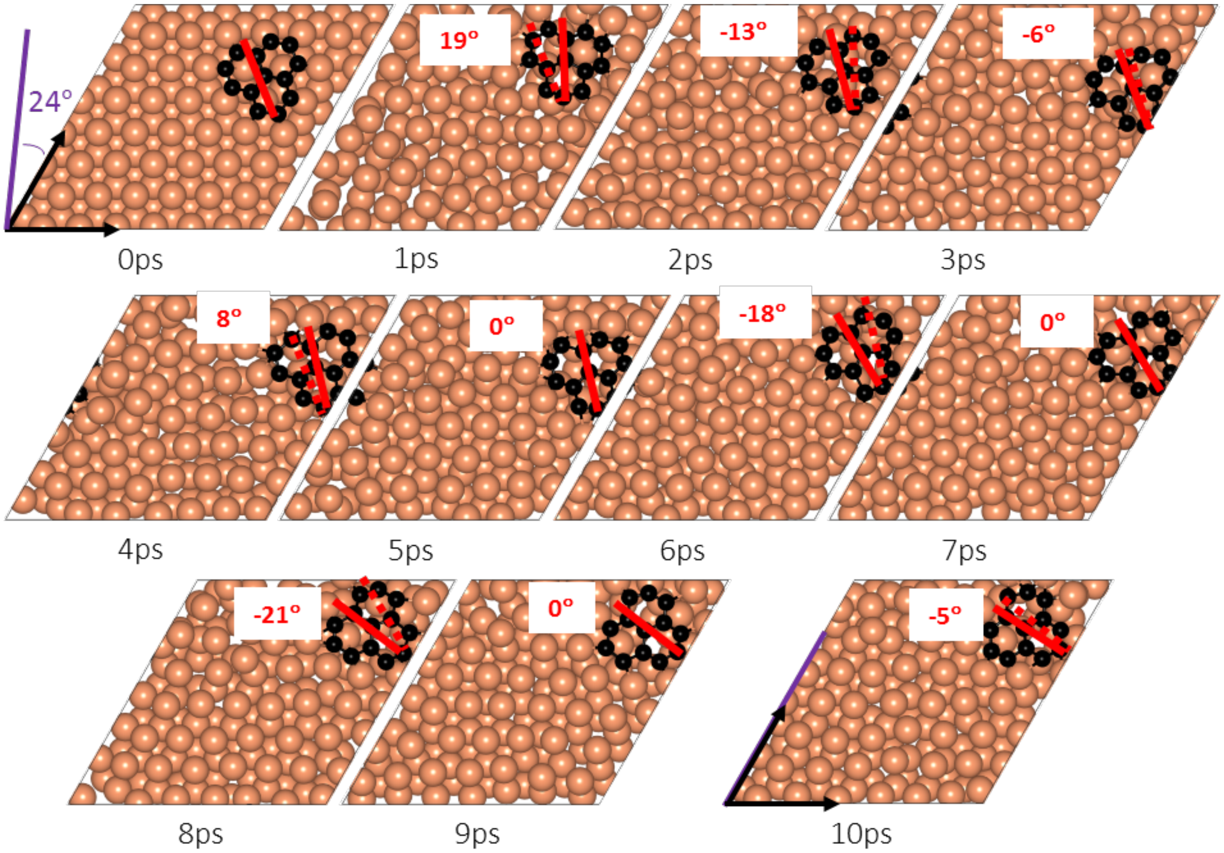

Figure S7. Evolution of a lone graphene fragment adsorbed on the Cu (111) surface at 1200K. Results are depicted every 1ps time. The initial orientation (24°) of the fragment with respect to Cu lattice is shown in purple at 0ps. The final orientation is shown at 10ps and is zero. The solid and dashed red lines follow the rotation of the fragment about its own axis. Negative values correspond to anti-clockwise rotation.

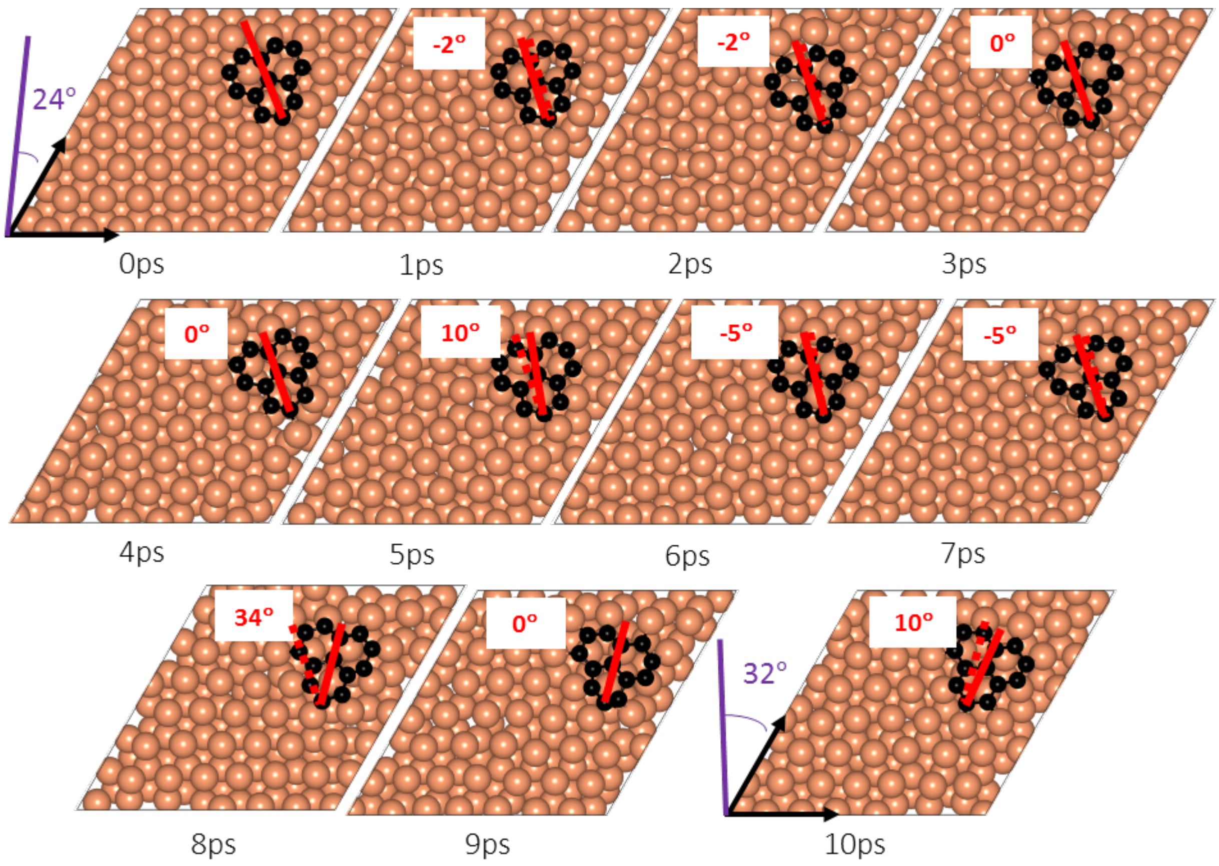

Figure S8. Evolution of a lone graphene fragment adsorbed on the Cu (111) surface at 900K. Results are depicted every 1ps time. The initial orientation (24°) of the fragment with respect to Cu lattice is shown in purple at 0ps. The final orientation is shown at 10ps and is 32°. The solid and dashed red lines follow the rotation of the fragment about its own axis. Negative values correspond to anti-clockwise rotation.

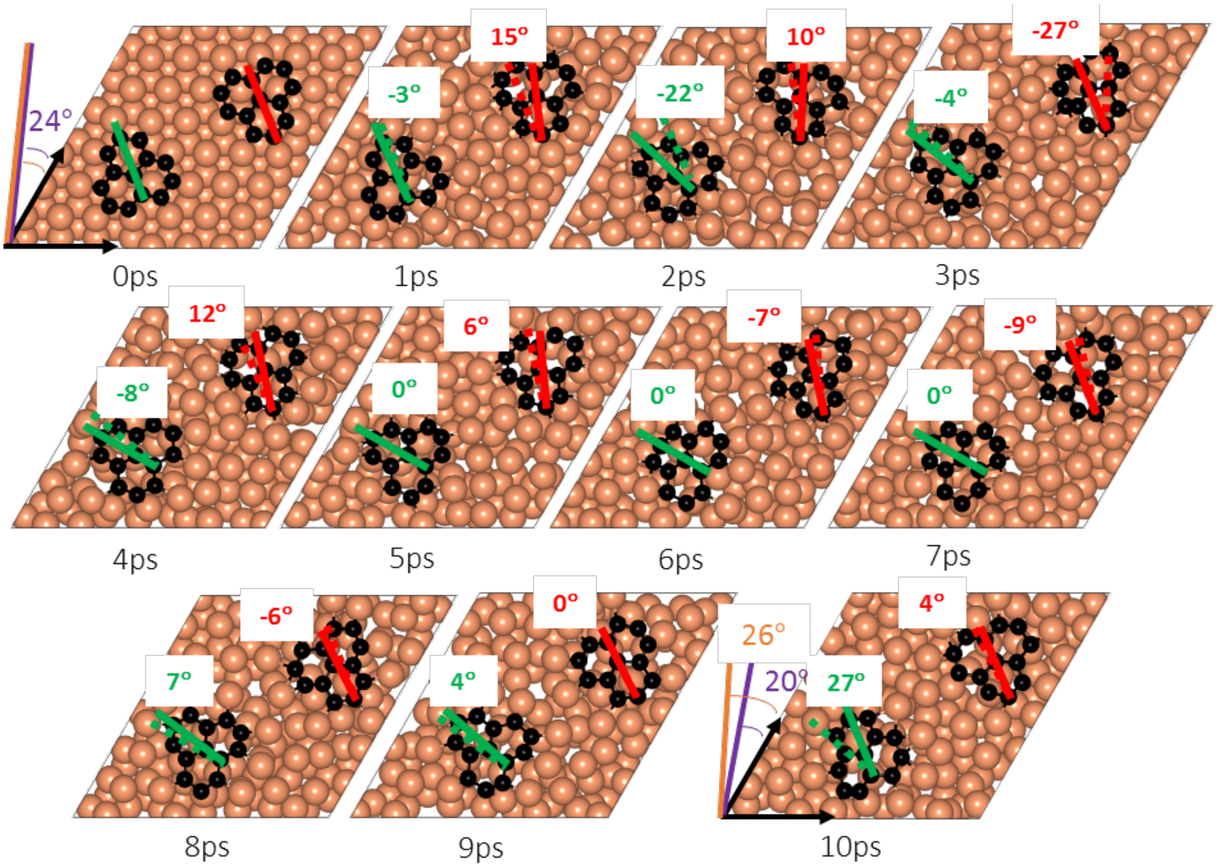

Figure S9. Evolution of a pair of graphene fragments adsorbed on the Cu (111) surface at 1200K. Results are depicted every 1ps time. The initial orientation (24°) of the fragment with respect to Cu lattice is shown in purple at 0ps. The final orientations are shown at 10ps and are 26° for the left fragment and 20° for the right. The solid and dashed red lines follow the rotation of the fragment about its own axis. Negative values correspond to anti-clockwise rotation.

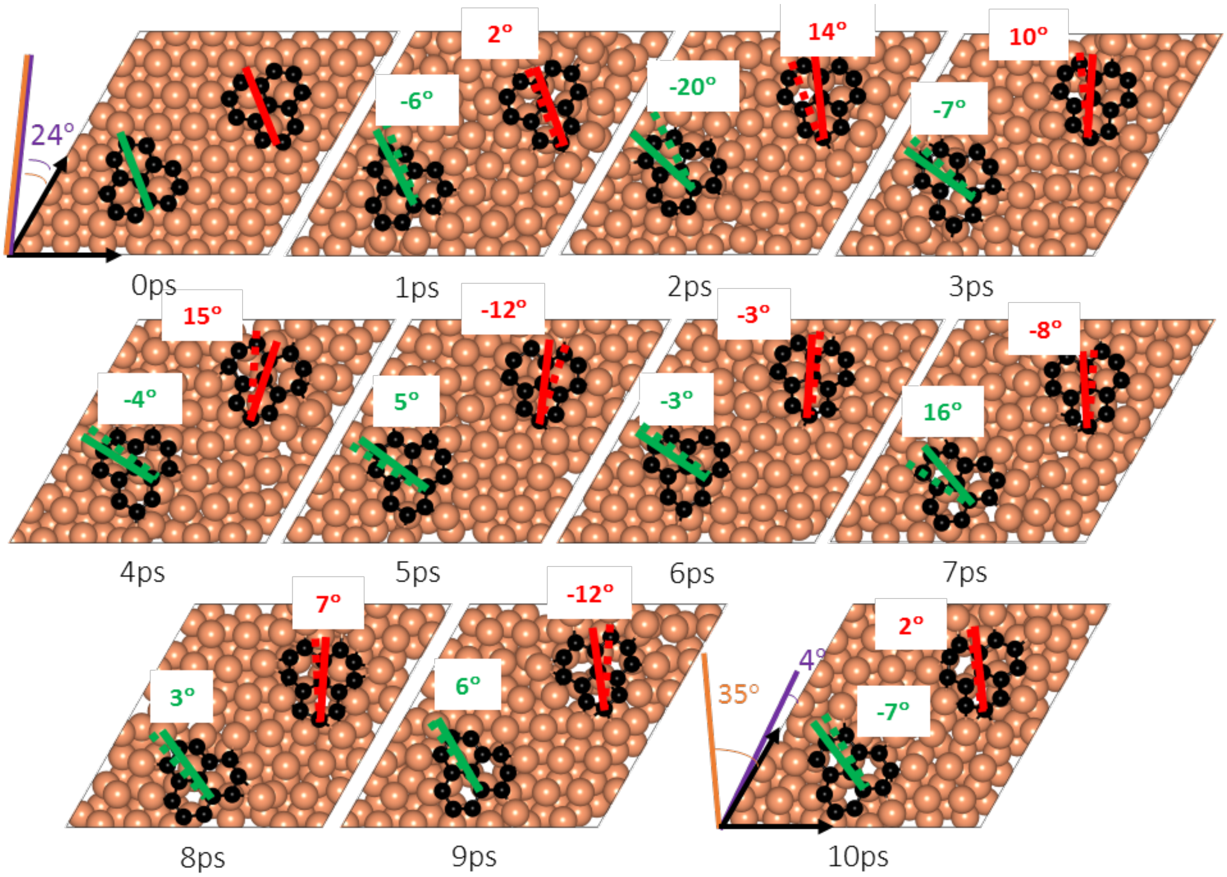

Figure S10. Evolution of a pair of graphene fragments adsorbed on the Cu (111) surface at 900K. Results are depicted every 1ps time. The initial orientation (24°) of the fragment with respect to Cu lattice is shown in purple at 0ps. The final orientations are shown at 10ps and are 35° for the left fragment and 4° for the right. The solid and dashed red lines follow the rotation of the fragment about its own axis. Negative values correspond to anti-clockwise rotation.

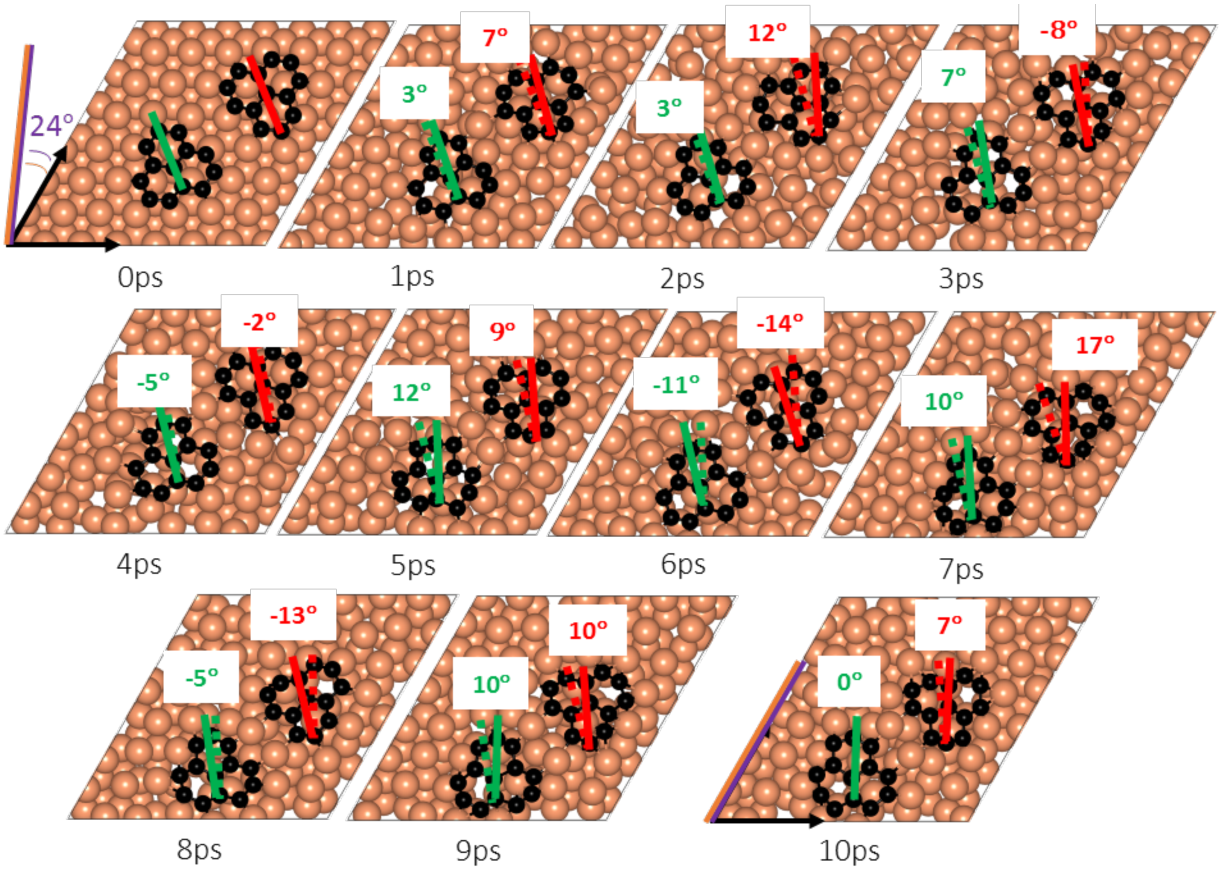

Figure S11. Evolution of a pair of close-proximity graphene fragments adsorbed on the Cu (111) surface at 1200K. Results are depicted every 1ps time. The initial orientation (24°) of the fragment with respect to Cu lattice is shown in purple at 0ps. The final orientations are shown at 10ps and are zero for both fragments. The solid and dashed red lines follow the rotation of the fragment about its own axis. Negative values correspond to anti-clockwise rotation.

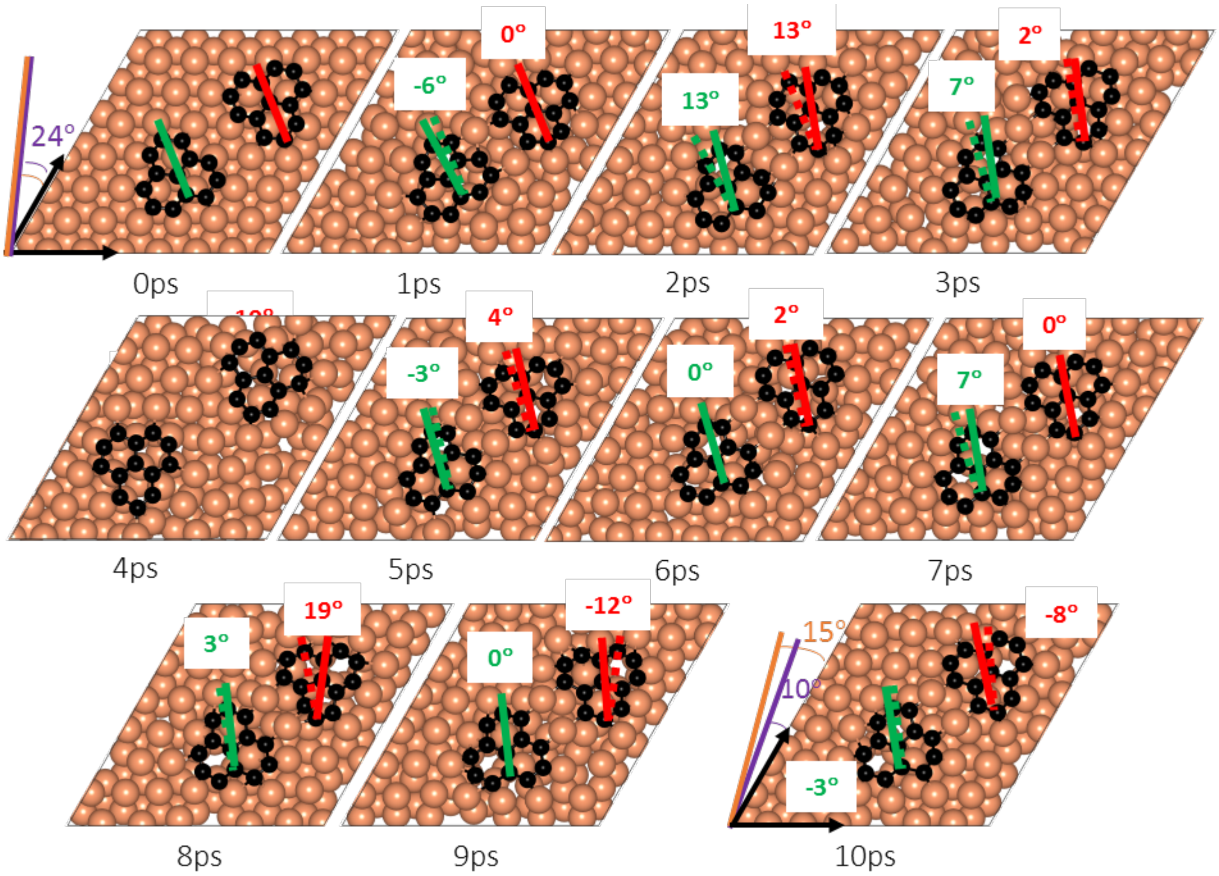

Figure S12. Evolution of a pair of close-proximity graphene fragments adsorbed on the Cu (111) surface at 900K. Results are depicted every 1ps time. The initial orientation (24°) of the fragment with respect to Cu lattice is shown in purple at 0ps. The final orientations are shown at 10ps and are 15° for left fragment and 10° for the right. The solid and dashed red lines follow the rotation of the fragment about its own axis. Negative values correspond to anti-clockwise rotation.

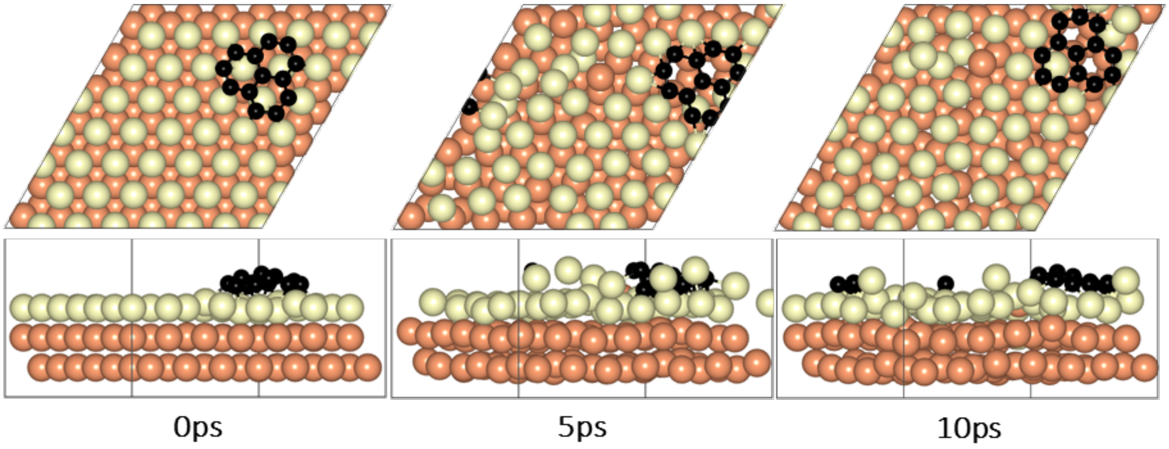

Figure S13. Surface reconstruction of the Cu slab in the presence of a graphene fragment taken from 5ps and 10ps of AIMD simulations performed at 1200K. Light green atoms depict surface Cu atoms.

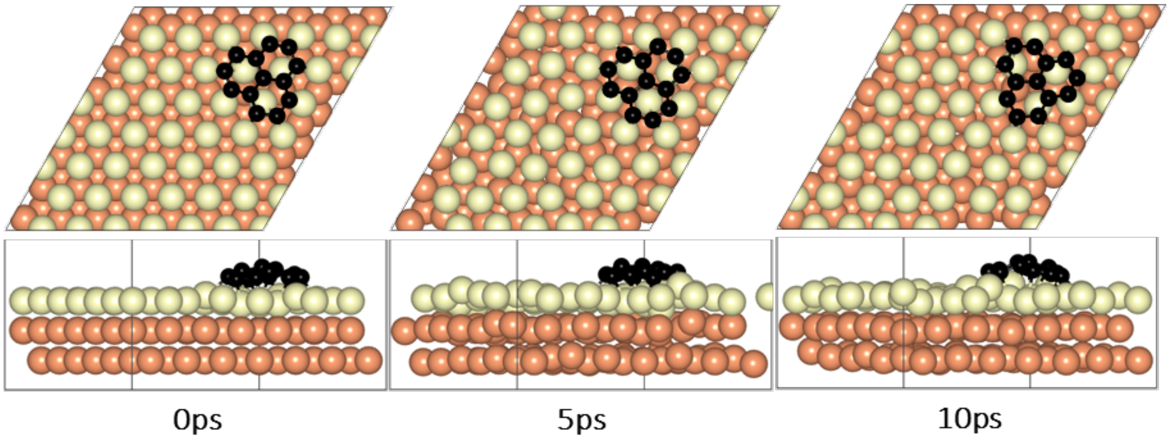

Figure S14. Surface reconstruction of the Cu slab in the presence of a graphene fragment taken from 5ps and 10ps of AIMD simulations performed at 900K. Light green atoms depict surface Cu atoms.

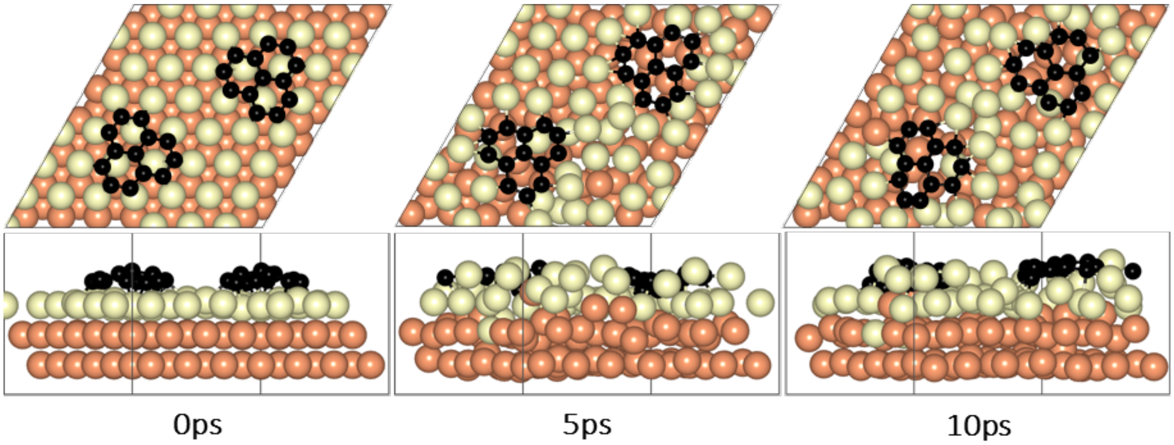

Figure S15. Surface reconstruction of the Cu slab in the presence of a pair of graphene fragments taken from 5ps and 10ps AIMD simulations performed at 1200K. Light green atoms depict surface Cu atoms.

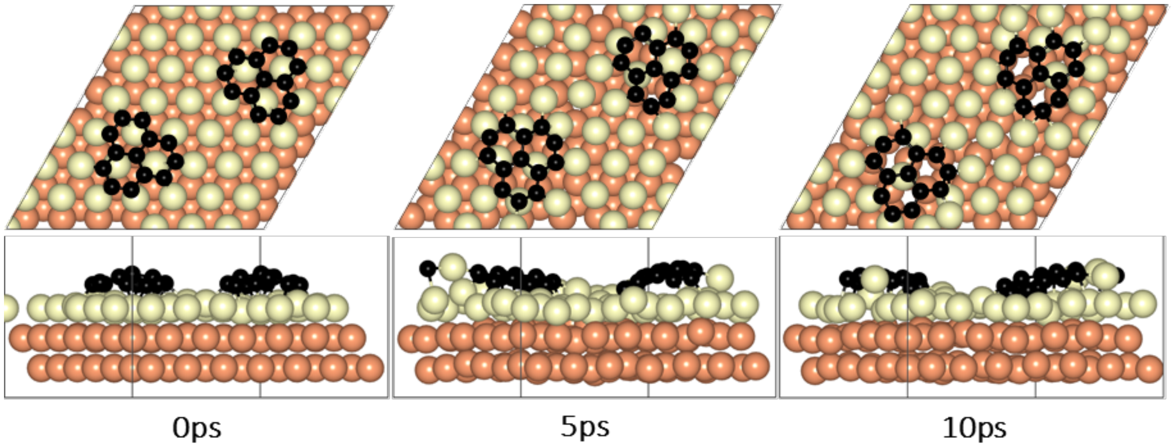

Figure S16. Surface reconstruction of the Cu slab in the presence of a pair of graphene fragments taken from 5ps and 10ps AIMD simulations performed at 900K. Light green atoms depict surface Cu atoms.

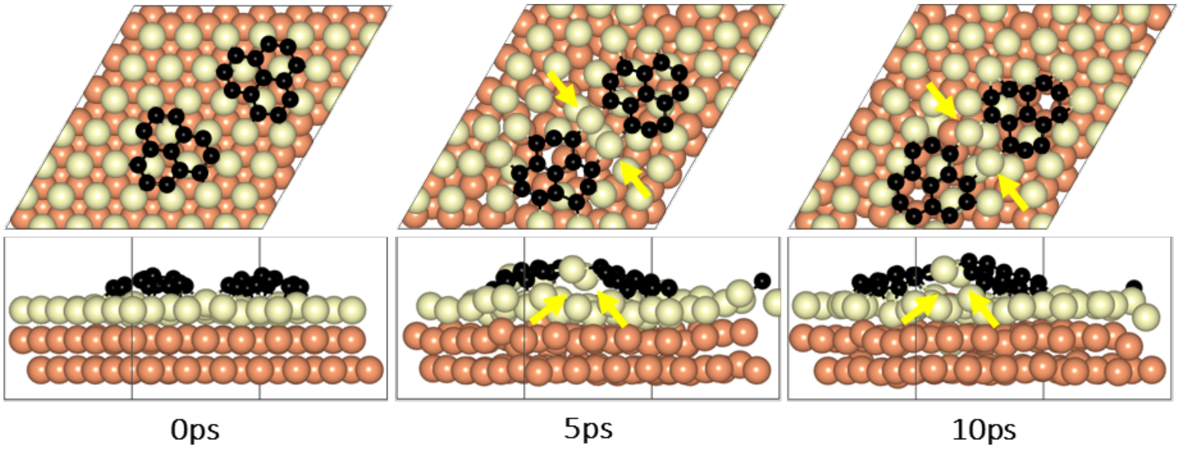

Figure S17. Surface reconstruction of the Cu slab in the presence of a pair of close-proximity graphene fragments taken from 5ps and 10ps of AIMD simulations performed at 1200K. Light green atoms depict surface Cu atoms.

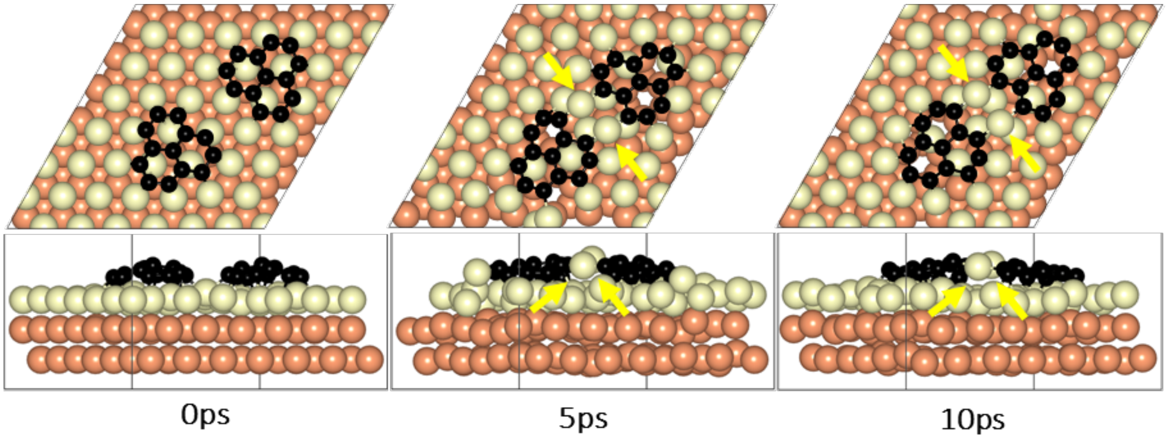

Figure S18. Surface reconstruction of the Cu slab in the presence of a pair of close-proximity graphene fragments taken from 5ps and 10ps of AIMD simulations performed at 900K. Light green atoms depict surface Cu atoms.
